# Radiation improves antitumor effect of immune checkpoint inhibitor in murine hepatocellular carcinoma model

**DOI:** 10.18632/oncotarget.17168

**Published:** 2017-04-17

**Authors:** Kyoung-Jin Kim, Ji-Hye Kim, Seo Jin Lee, Eun-Jung Lee, Eui-Cheol Shin, Jinsil Seong

**Affiliations:** ^1^ Department of Radiation Oncology, Yonsei Cancer Center, Yonsei University College of Medicine, Seoul, Republic of Korea; ^2^ Laboratory of Immunology and Infectious Diseases, Graduate School of Medical Science and Engineering, Korea Advanced Institute of Science and Techonology, Daejeon, Republic of Korea

**Keywords:** hepatocellular carcinoma, anti-PD-L1, radiation, combination therapy, antitumor effect

## Abstract

**Background & aims:**

Although immunotherapy has emerged as an attractive therapy for refractory cancers, its limited efficacy in hepatocellular carcinoma (HCC) suggests the need for a combination strategy that can either enhance or complement therapeutic effect. We investigated whether combination of immune checkpoint blockade (ICB) and radiation could enhance antitumor effect in a murine HCC model.

**Methods:**

Using murine HCC, HCa-1, the effect of radiation on programmed death-ligand1 (PD-L1) expression was determined by real-time PCR, flow cytometry, and western blotting. Signaling pathways involved in altered PD-L1 expression were examined. Tumor growth and survival rate were evaluated for a combination of anti-PD-L1 and radiation. Immunological parameters in the tumor were assessed using flow cytometry and histological study.

**Results:**

Radiation upregulated PD-L1 expression in tumor cells through IFN-γ/STAT3 signaling, which could facilitate therapeutic action of anti-PD-L1. Combination of anti-PD-L1 and radiation significantly suppressed tumor growth compared to treatment with anti-PD-L1 alone or radiation alone group (*P<*0.01). Survival was significantly improved in the combination group compared to anti-PD-L1 alone or radiation alone group (7-week survival rate; 90% vs. 0% or 30%, respectively, *P<*0.001). The underlying mechanism involved increasing apoptosis, decreasing tumor cell proliferation, as well as restoration of CD8^+^ T cell functions.

**Conclusions:**

The combination of anti-PD-L1 and radiation significantly improved the antitumor effect shown in tumor growth delay as well as in survival, supporting a novel combination strategy of immunoradiotherapy in HCC.

## INTRODUCTION

Hepatocellular carcinoma (HCC) is one of the most common malignancies worldwide with a high mortality rate. Particularly, advanced HCC patients have a poor prognosis with a 5-year survival rate <5% and a high recurrence rate [1, 2]. Although sorafenib, a multityrosine kinase inhibitor, has been in used in clinical practice as the standard of care, the median survival stays only a few months [3–5] since cancer tends to acquire drug resistance as well as invasion and intra- and extra-hepatic metastasis after treatment [6, 7]. Hence, therapeutic approaches are necessary for not only powerful local treatment of tumor, but also to induce systemic antitumor effects [8].

Recently, immunotherapy has emerged as an effective and promising treatment for cancers that were refractory to conventional treatment. Recent advances in the understanding of tumor biology and immune checkpoint molecules have provided novel therapeutic strategies using immune checkpoint blockade (ICB). Several studies have shown that the blockade of cytotoxic T lymphocyte-associated antigen-4 (CTLA-4) or programmed death-1 (PD-1) resulted in significant antitumor effect and prolongation of survival [9–13]. However, modest response rates have indicated that its efficacy appears to be insufficient [11, 14]. Therefore, development of combination therapies seems necessary, which can not only provide direct tumoricidal effect but also enhance the antitumor effect of immunotherapy.

Radiation therapy (RT) has long been utilized as one of major cancer modality. RT results in direct tumor cell death and induces tumor-specific immune response [15, 16]. It can also modify the local tumor microenvironment, so that improve immune cell trafficking into the tumor site [17]. Recent preclinical and clinical studies show that RT induces immunogenic modulation of surviving tumor cells involving upregulation of tumor associated antigens, death receptor, and cell surface molecules, suggesting that RT can promote tumor susceptibility to cytotoxic T lymphocyte-mediated tumor control [18–23].

Several preclinical studies showed that radiation combined with ICB enhanced local tumor control and survival rates in breast cancer, colon cancer, and melanoma [24–26], providing evidences of the synergistic effect in the combination of ICB and radiation in solid tumor. In HCC, radiotherapy is still not a standard of care according to some guidelines (BCLC guideline, Llovet et al, Seminars in Liver Disease, 1999), while it is one of many options in other guidelines (NCCN guideline, 2016). Consequently, investigation of combining radiotherapy with ICB has been seriously limited, despite unsatisfactory result of ICB alone in HCC [27].

We hypothesized that if radiation could induce programmed death-ligand1 (PD-L1) expression in the tumor, the antitumor effect of PD-L1 blockade would be augmented along with the antitumor effect of radiation per se. In this study, we investigated whether radiation could enhance PD-L1 expression in tumor cells, which may provide an effective milieu for anti-PD-L1 binding to tumor cells, as well as its impact on tumor growth suppression and survival, using murine HCC syngeneic to C3H/HeN mice as a preclinical model for HCC.

## RESULTS

### Radiation upregulated the expression of PD-L1 in murine HCC cells

**Figure 1:**
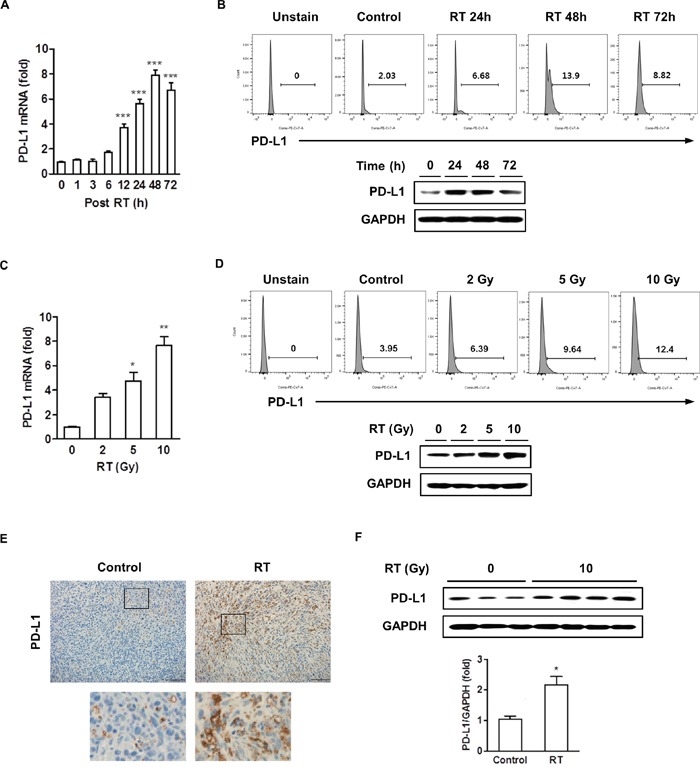
Radiation increased the expression of PD-L1 *in vitro* and *in vivo* HCa-1 cells were treated with 10 Gy radiation for the indicated times. Increased expression of PD-L1 was measured by **(A)** real-time PCR, **(B)** flow cytometry and western blotting. HCa-1 cells received 0, 2, 5, or 10 Gy radiations and were cultured for 2 days (*** *P*<0.001, one-way ANOVA). PD-L1 expression was analyzed using **(C)** real-time PCR, **(D)** flow cytometry and western blotting. (* *P*<0.05, ** *P*<0.01, one-way ANOVA). GAPDH was the loading control. Data are from three independent experiments. The effect of radiation on PD-L1 expression *in vivo* was measured; mice implanted with HCa-1 cells were treated with 10 Gy radiation and protein expressions were assessed in tumors, obtained after 7 days, by **(E)** IHC staining (original magnification 200×, scale bar = 100 μm) and **(F)** western blotting (* *P*<0.05, student *t* test). Data are from two independent experiments (n=3 or 4 per group).

We first assessed the *in vitro* effect of radiation on PD-L1 expression in murine HCC (HCa-1). The change of PD-L1 mRNA expression after radiation was determined by real-time PCR and PD-L1 protein expression was determined by flow cytometry and western blotting. Figure 1A shows the time-course of radiation-induced PD-L1 mRNA expression. PD-L1 mRNA expression increased slightly at 6 h after radiation, their maximal value was achieved between 24-48 h, and the expression declined thereafter. The PD-L1 protein expression pattern was similar to the mRNA expression levels (Figure 1B). We also tested for radiation-induced increase in PD-L1 expression in other HCC cell lines, and found that PD-L1 protein expression increased in murine cell lines (MIH2 and Hepa 1-6) and human cell lines (Huh7 and HepG2) (Supplementary Figure 1). To assess the influence of radiation in inducing PD-L1 expression in tumor cells, we conducted a radiation dose-response test, and the results revealed that the expression of PD-L1 was upregulated in a dose-dependent manner (Figure 1C, 1D). Therefore, all of the subsequent experiments were tested with 10 Gy radiation. We also examined the *in vivo* effect of radiation on PD-L1 expression by immunohistochemistry (IHC) and western blotting. HCa-1 cells (1 × 10^6^) were inoculated intramuscularly into the right thighs of mice, and tumors were irradiated with a single dose of 10 Gy when the tumor reached to 8 mm in mean diameter. To examine the PD-L1 expression, tumor samples were harvested 7 days after radiation. Tumor sections were stained with PD-L1 antibody for IHC and tumor cell lysate was isolated for western blotting. As shown in Figure 1E and 1F, radiation increased PD-L1 expression in the tumor. In orthotopic model, radiation resulted in increased upregulation of PD-L1 expression in the tumor tissue, without affecting the normal liver adjacent to the tumor (Supplementary Figure 2). These results collectively suggest that radiation upregulates PD-L1 expression in HCC cells in both, a time- and dose-dependent manner.

### Radiation upregulated IFN-γ and TNF-α expression and IFN-γ was involved in radiation-induced PD-L1 expression in HCC cells

**Figure 2:**
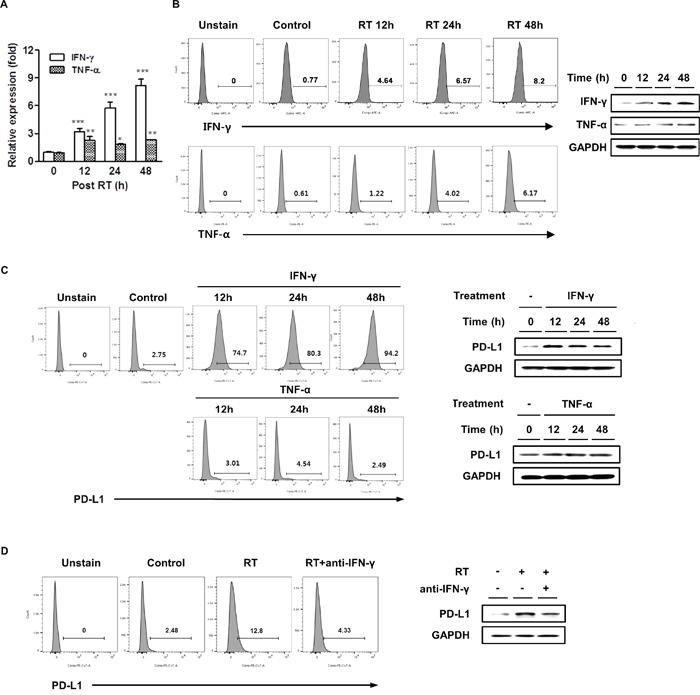
Radiation increased IFN-γ and TNF-α expressions and IFN-γ was correlated with radiation-induced PD-L1 expression in HCC cells HCa-1 cells were treated with 10 Gy radiation. IFN-γ and TNF-α expressions were measured by **(A)** real-time PCR, **(B)** flow cytometry and western blotting (* *P*<0.05, ** *P*<0.01, *** *P*<0.001, one-way ANOVA). GAPDH was the loading control. HCa-1 cells were treated with IFN-γ (20 ng/ml) or TNF-α (20 ng/ml) for the indicated times. PD-L1 expression was analyzed by **(C)** flow cytometry and western blotting. Anti-IFN-γ antibody (10 μg/ml) was added 30 min before radiation and PD-L1 expression was measured by **(D)** flow cytometry and western blotting. Data are from three independent experiments.

In several cancer cells, upregulation of PD-L1 expression is strongly associated with a Toll-like receptor or the IFN-γ signaling pathway [28, 29]. Radiation can cause an inflammatory milieu by inducing the release of proinflammatory cytokines, including IFN-γ, TNF-α, and interleukin-6 [18]. Therefore, we investigated possible tumor-derived cytokines induced by radiation that contributed to the upregulation of PD-L1 expression. HCa-1 cells were cultured for 48 h after radiation, then the IFN-γ and TNF-α expression was determined by real-time PCR, flow cytometry, and western blotting. Figure 2A shows that radiation induced both, IFN-γ and TNF-α mRNAs; however, only the induction of IFN-γ mRNA levels positively correlated to PD-L1 mRNA expression (Figure 1A). We also examined the effect of radiation on IFN-γ and TNF-α protein expression by flow cytometry and western blotting, the results demonstrated that radiation increased these protein expressions with kinetics similar to those observed for the mRNA expression (Figure 2B). We next examined the role of these cytokines on PD-L1 expression in HCa-1 cells. Treatment of recombinant IFN-γ resulted in increased upregulation of the surface PD-L1 expression in HCa-1 cells, while treatment of recombinant TNF-α had little effect on PD-L1 expression (Figure 2C).

### PD-L1 expression decreased after IFN-γ blockade in HCC cells

The above results suggest that radiation could upregulate PD-L1 expression via IFN-γ production. To understand whether radiation upregulated the PD-L1 expression in HCa-1 cells via IFN-γ signaling, blocking experiments were performed. We observed the effect of IFN-γ inhibition on the expression of PD-L1. Pre-treatment with the anti-IFN-γ neutralizing antibody resulted in significant inhibition in radiation-induced upregulation of PD-L1 expression, as assessed by flow cytometry and western blotting (Figure 2D). These data indicate that IFN-γ plays an important role in radiation-induced PD-L1 expression.

### IFN-γ upregulated the expression and phosphorylation of STAT3 in HCC cells

**Figure 3:**
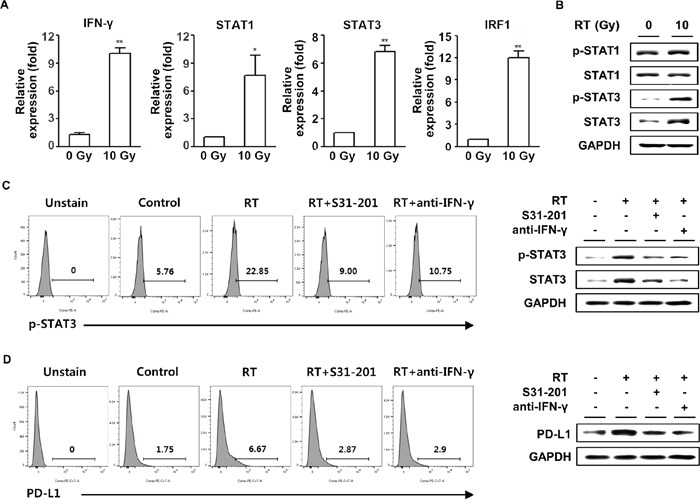
Upregulation of PD-L1 expression in tumor cells following radiation was IFN-γ-dependent HCa-1 cells were treated with 10 Gy radiation for 24 h and cells were harvested for the measurement of mRNA expression by **(A)** real-time PCR (* *P*<0.05, ** *P*<0.01, one-way ANOVA). Phosphorylation and protein expression of STAT1 and STAT3 were measured by western blotting **(B)**. S31-201 (50 μM) or anti-IFN-γ (10 μg/ml) was added 30 min before 10 Gy radiation. The phosphorylation and protein expression of STAT3 **(C)** and expression of PD-L1 **(D)** was measured by flow cytometry and western blotting. GAPDH was the loading control. Data are from three independent experiments.

Lee *et al*. showed that Janus activated kinase/STAT/interferon regulatory factor-1 (IRF-1) signaling pathway plays a critical role in the upregulation of PD-L1 expression in response to IFN-γ in human lung cancer cell lines [30]. Therefore, we examined the expressions of the IFN-γ downstream signaling molecules in HCa-1 cells following radiation. Using real-time PCR, increased mRNA levels of STAT1 and STAT3 were observed. In addition, the main target of IFN-γ, IRF-1, was also increased in HCa-1 cells exposed to radiation (Figure 3A). Radiation enhanced STAT3 protein expression and phosphorylation, whereas no changes were found in phosphorylation and protein expression of STAT1 (Figure 3B). Thus among these molecules, we focused on the role of STAT3 in the radiation-induced upregulation of PD-L1 expression. As shown in Figure 3C, radiation resulted in the increase in phosphorylation and protein expression of STAT3, which were inhibited by pre-treatment with the STAT inhibitor, S31-201, or the IFN-γ blocking antibody, anti-IFN-γ. We next examined PD-L1 expression in STAT3 or IFN-γ-inhibited HCa-1 cells. Blockade of IFN-γ or STAT3 significantly inhibited the PD-L1 expression induced by radiation (Figure 3D). In addition, we performed knock-down experiments to confirm the role of STAT3 in radiation-induced PD-L1 expression. Radiation-induced PD-L1 expression was attenuated by transfection of STAT3 siRNA, while transfection of STAT1 siRNA had no effect on PD-L1 expression (Supplementary Figure 3). These results suggest that radiation-induced IFN-γ production, which increased expression and phosphorylation of STAT3, and ultimately increased PD-L1 expression in tumor cells.

### Combination of anti-PD-L1 and radiation treatments increased antitumor effect of radiation in murine HCC model

**Figure 4:**
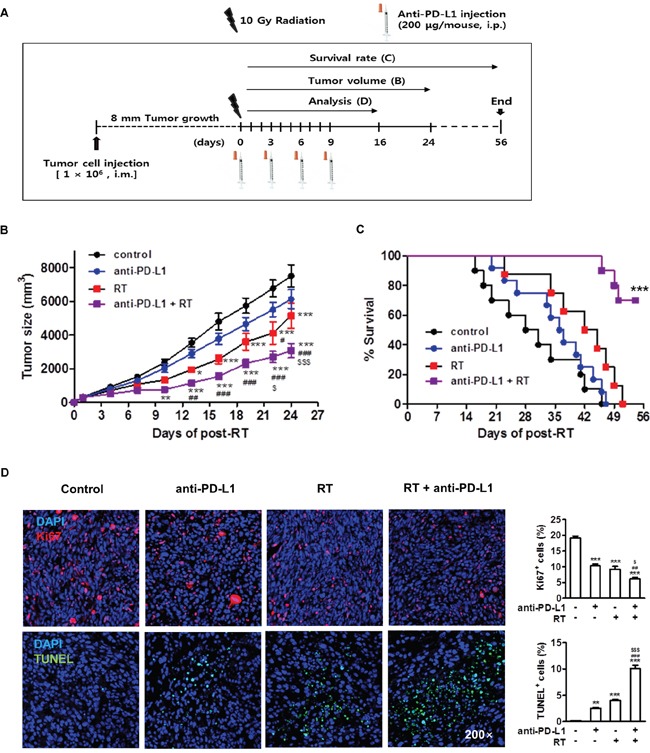
Combination of anti-PD-L1 and radiation increased antitumor effect in murine HCC model **(A)** Experimental design for the combined treatment with anti-PD-L1 and radiation. **(B)** Tumor growth was measured twice per week following radiation and tumor size was shown as mean size ± SE. * *P*<0.05; ** *P*<0.01; *** *P*<0.001 compared with the control group; # *P*<0.05; ## *P*<0.01; ### *P*<0.001 compared with the anti-PD-L1 group; $ *P*<0.05; $$$ *P*<0.001 compared with the RT group (two-way ANOVA) **(C)** Survival rate was recorded as the percentage of surviving mice on a given day (*** *P*<0.001, log-rank (Mantel-Cox) test). Seven days after the last treatment, the tumors were removed. **(D)** Immunohistochemical analysis of the tumors stained with Ki67 and TUNEL (left, original magnification 200×). Quantitative analysis of Ki67^+^ and TUNEL^+^ cells among the indicated group (right). Data are from three independent experiments (n=5 per group). ** *P*<0.01; *** *P*<0.001 compared with the control group; ## *P*<0.01; ### *P*<0.001 compared with the anti-PD-L1 group; $ *P*<0.05; $$$ *P*<0.001 compared with the RT group (one-way ANOVA).

The above data showed that radiation led to enhancement of tumor cell PD-L1 expression *in vivo* and *in vitro*. Expression of PD-L1 may allow tumor cells to evade the host immune response and attenuate the efficacy of the antitumor immune response [31]. We next examined whether radiation could improve effect of anti-PD-L1 treatment *in vivo*. HCa-1 cells (1 × 10^6^) were inoculated intramuscularly into the right thighs of mice, and treatment was started when the tumor reached to 8 mm in mean diameter. Right thighs of mice were irradiated with 10 Gy in 1 fraction. For the PD-L1 blockade experiment, anti-PD-L1 (10 mg/kg) was given a total of 4 injections in 3-day intervals after radiation (Figure 4A). The antitumor effects of the combination therapy were determined using the tumor growth delay and survival rate. Tumor growth suppression was not significant in the anti-PD-L1 alone group (*P*>0.05), but was significant in the radiation alone group compared with the control group (*P*<0.05). The lack of tumor growth suppression in anti-PD-L1 alone could have resulted from low baseline level of PD-L1 in HCa-1 cells. Combination group significantly suppressed tumor growth compared with anti-PD-L1 or radiation alone group, respectively (*P*<0.01 and *P*<0.05, respectively) (Figure 4B; on day 24, control, anti-PD-L1, radiation, and combination group: 7512 ± 661 mm^3^, 5149.7 ± 752.2 mm^3^, 6130.1 ± 559.2 mm^3^, and 3080.122 ± 447.9 mm^3^, respectively).

Survival was significantly improved in the combination group compared to anti-PD-L1 or radiation alone group (7-week survival rate; 90% vs. 0% or 30%, respectively). After all mice in the other experimental groups had died, 70% of mice still survived in the combination group (*P*<0.001) (Figure 4C).

We next examined the mechanism underlying the significant regression of HCa-1 tumors exhibited in the combination treatment. The rates of cell proliferation and apoptosis in tumor tissues from each group were analyzed by IHC staining for Ki67 and terminal deoxynucleotidyl transfer dUTP nick end labeling (TUNEL), respectively. The combination group exhibited significantly decreased tumor cell proliferation compared with the anti-PD-L1 or with the radiation alone group. The level of apoptosis was increased in the combination group compared with the anti-PD-L1 or with the radiation alone group (Figure 4D), which might have contributed to the suppression of tumor growth.

### Combination of anti-PD-L1 and radiation restored CD8^+^ T cell function

**Figure 5:**
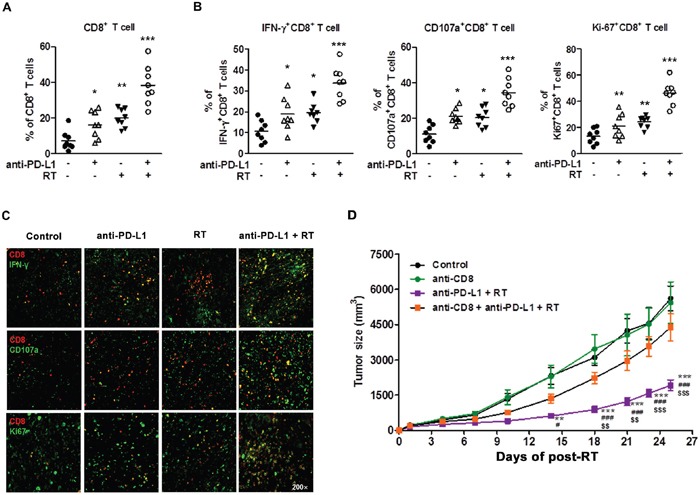
Combination of anti-PD-L1 and radiation increased infiltration of CD8^+^ T cells into the tumor and restored CD8^+^ T cell functions HCa-1-bearing mice were treated with anti-PD-L1 and radiation, as described in Figure 4A. Seven days after the last treatment, TILs were isolated for flow cytometry or tumor tissues were harvested for co-immunofluorescence staining. Next, the staining for CD8, IFN-γ, CD107a, and Ki-67 were performed. Frequency of **(A)** CD8^+^ T cells, **(B)** IFN-γ^+^CD8^+^ T cells, CD107a^+^CD8^+^ T cells, and Ki67^+^CD8^+^ T cells in TILs are shown. The horizontal bars indicate mean values (* *P*<0.05, ** *P*< 0.01, *** *P*<0.001, one-way ANOVA). **(C)** Representative images of CD8, IFN-γ, CD107a, and Ki67 in tumors were confirmed by co-immunofluorescence staining (original magnification 200×). Data are from two independent experiments (n=4 per group). **(D)** Tumor growth inhibition using combination therapy mediated by CD8^+^ T cells. Starting from one day before radiation, 12.5 mg/kg of CD8^+^ depletion antibody was administrated i.p. every three days for a total of four times. Tumor growth was measured twice per week following radiation, and tumor size was shown as mean size ± SE. ** *P*<0.01; *** *P*<0.001 compared with the control group; # *P*<0.05; ### *P*<0.001 compared with the anti-CD8 group; $$ *P*<0.01; $$$ *P*<0.001 compared with the anti-CD8 plus combination group (two-way ANOVA).

Enhanced PD-L1 expression can promote T cell exhaustion, which causes T cell dysfunction. Exhausted T cells upregulate inhibitory receptors, including PD-1 and CTLA-4, which leads to the loss of their effector function [32]. CD8^+^ tumor infiltrating lymphocytes (TILs) are known to play a key role in antitumor effect and PD-1-expressing CD8^+^ TILs are involved in tumor-associated immunosuppression [33]. To investigate whether a combination of anti-PD-L1 and radiation could reverse CD8^+^ TIL functions, we performed flow cytometry analysis using TILs isolated from HCa-1-bearing mice. The percentage of CD8^+^ T cells was significantly increased in the combination group, while anti-PD-L1 and radiation alone groups showed a modest increase in CD8^+^ T cells (Figure 5A; control, anti-PD-L1, radiation, and combination group: 7.17 ± 1.91%, 20.19 ± 2.09%, 17.13 ± 3.55%, and 38.36 ± 3.87%, respectively). Next, we examined the ability to reverse CD8^+^ T cell effector functions by measuring IFN-γ production, degranulation (CD107a), and cell proliferation (Ki67). As shown in Figure 5B, combination group significantly increased the IFN-γ^+^CD8^+^T cells, CD107a^+^CD8^+^ T cells, and Ki67^+^CD8^+^ T cells, indicating an increased effector cell cytotoxicity and proliferation (combination vs. control group; 32.03 ± 2.29% vs. 10.57 ± 2.86%, 46.02 ±3.16% vs. 18.02 ± 3.24%, 30.52 ± 3.97% vs. 14.39 ± 4.86%, respectively). These results were confirmed at the protein level by IHC (Figure 5C).

These data suggest that an infiltration of effector CD8^+^ T cells may be a major mechanism mediating the combined treatment effect. To investigate the importance of CD8^+^ T cells in combination therapy, we depleted mice of CD8^+^ T cells before treatment with combination therapy. Depletion of CD8^+^ T cells were performed by repeated injections of anti-CD8 antibody over the 3-day interval. When the mice were depleted of CD8^+^ T cells, effectiveness of combination therapy was abrogated, resulting in rapid tumor outgrowth (Figure 5D). These results show that CD8^+^ T cells were essential for therapeutic effect of combination therapy. Taken together, our data indicate that combination of anti-PD-L1 and radiation can restore CD8^+^ T cells to expand and regain their effector functions, which contribute to the therapeutic antitumor effect.

## DISCUSSION

In this study, we showed that combination of anti-PD-L1 with radiation enhanced the antitumor effects of radiation on murine HCC. This conclusion was based upon the following findings: (i) radiation upregulated PD-L1 expression in tumor cells in a time- and dose-dependent manner, (ii) radiation activated STAT3 through the production of IFN-γ, which led to PD-L1 upregulation in tumor cells, and (iii) the efficacy of radiation could be increased by combination with anti-PD-L1 antibody, leading to the abrogation of exhausted CD8^+^ T cells in a murine HCC model.

Although immunotherapy has emerged as a novel cancer treatment, its modest response rate particularly in HCC throws an issue of how to escalate its antitumor effect. Tumors with a high mutation burden, such as melanoma and non-small cell lung cancer, showed clinical benefits to immune checkpoint therapy. High frequency of non-synonymous mutation, which results in the formation of neo-antigens that can be recognized by immune cells, has been reported to be successful for cancer immune checkpoint therapy [34]. In addition, mutation burden serves as the predictive biomarker for response to immune checkpoint inhibitors [35, 36]. ICB, anti-CTLA-4, anti-PD-1, and anti-PD-L1 have been approved for treatment of melanoma and non-small cell lung cancer. Clinical outcome of anti-PD-1 antibody treatment was shown in phase I/II trials for patients with HCC, with a response rate of 19% [37]. Since mutational burden of HCC is lower than melanoma and lung cancer [34], the efficacy of anti-PD-1 treatment does not seems to be sufficient to elicit durable clinical benefit. It has been suggested that ICB needs to be combined with other therapies that can either induce tumor mutation burden or enhance the antitumor effect of immunotherapy, and RT has been suggested as a possible combining agent to ICB therapy [15].

Radiation induces not only direct tumor cell killing but also phenotype alteration of tumor cells, including MHC-I, carcinoembryonic antigen, mucin-1, Fas, and ICAM-1, which may affect tumor immunogenicity [18–23]. Radiation can release damage-associated molecular patterns, which can lead to recruitment of dendritic cells and stimulation of tumor antigen-specific T cell response [38, 39]. Although RT led to antitumor effect by inflammatory response, immune-escape often occurs and tumors may acquire resistance which promotes relapse [38, 39]. HCC also acquires frequent resistance to RT, and has resulted in recurrence [40]. One of the mechanisms of radio-resistance was upregulated PD-L1 in tumor microenvironment [24, 41]. PD-L1/PD-1 axis plays a key role in escape of host immune response by inducing T cell exhaustion [42]. Namely RT alone does not serve as a sufficient anti-tumor immune response. To overcome these limitations, novel therapies are required as the best way to reduce the rate of tumor recurrence in HCC. In a study by Shi and colleagues [43], increased PD-1 expression in circulating and tumor-infiltrating CD8^+^ T cells were associated with poor disease progression, such as shorter disease-free survival in HCC. These data suggest that blocking of PD-1/PD-L1 interaction may increase anti-tumor effect in HCC patients. For this reason, we focused on blocking the PD-1/PD-L1 pathway using anti-PD-L1 antibody in treatment of HCC.

In this study, we also found that radiation increased PD-L1 mRNA and protein expressions in HCa-1 cells in time- and dose- dependent manners (Figure 1). Although expression of PD-L1 was known to be induced in response to the proinflammatoy cytokine in many types of tumor [44], the underlying mechanism by which radiation upregulated PD-L1 expression in HCC remains unclear. In the present study, radiation significantly increased both IFN-γ and TNF-α production, but only radiation-induced IFN-γ showed the similar kinetics of radiation-induced PD-L1 expression (Figure 2A and 2B). In addition, PD-L1 expression was increased following treatment with IFN-γ in HCa-1 cells (Figure 2C), which is supported by a similar report from Liu *et al* [45]. These results indicate that PD-L1 expression could be regulated by radiation. Activation of several signaling pathways including IFN-γ, PI3K, STAT3, MAPK, and NF-κB are involved in upregulation of PD-L1 expression in various tumors [28, 46–48]. To investigate the underlying mechanism of PD-L1 upregulation in radiated-HCa-1 cells, we examined the IFN-γ/STAT3 signaling, as STAT3 activation can induce PD-L1 expression and STAT3 is one of the IFN-γ downstream signaling molecules [49, 50]. We found that radiation enhanced phosphorylation and expression of STAT3 as well as IFN-γ production, which might increase PD-L1 production in HCa-1 cells. Interestingly, treatment with an anti-IFN-γ antibody and a STAT3 inhibitor reduced PD-L1 expression in radiated-HCa-1 cells. These results suggest that the IFN-γ/STAT3 signaling pathway is closely related to the radiation-induced upregulation of PD-L1 in HCa-1 cells (Figure 3).

Several clinical studies showed that PD-L1 expression in tumors correlates with the high response rate of anti-PD-1 and anti-PD-L1 treatment [51]. In our study, radiation increased the induction of PD-L1 in tumors (Figure 1), making them susceptible to anti-PD-L1 treatment, which provides a strong evidence for investigation of radiation combined with anti-PD-L1 *in vivo*. We further demonstrated whether combination of anti-PD-L1 and radiation enhances the antitumor effect in a murine HCC model. Combination of anti-PD-L1 and radiation resulted in reduction of tumor growth and improved the survival rate when compared to treatment with either anti-PD-L1 or radiation (Figure 4). These therapeutic effects were associated with the induction of CD8^+^ T cell infiltration in the tumor, and reactivation and restoration of CD8^+^ T cell functions by blocking of PD-1/PD-L1 signaling (Figure 5). Taken together, our data indicate that combination of anti-PD-L1 and radiation can restore CD8 T^+^ cells to expand and regain their effector functions, which contribute to the therapeutic antitumor effect.

Zeng *et al*. reported that a combination therapy of anti-PD-1 antibody and radiation increases long term survival by induction of CD8^+^ T cells infiltration and reduction of regulatory T cells in a glioma animal model, which has a low mutational burden [52]. These reports, together with our data provide a support for a clinical strategy to induce a strong antitumor effect using anti-PD-L1 in combination with radiation.

**Figure 6:**
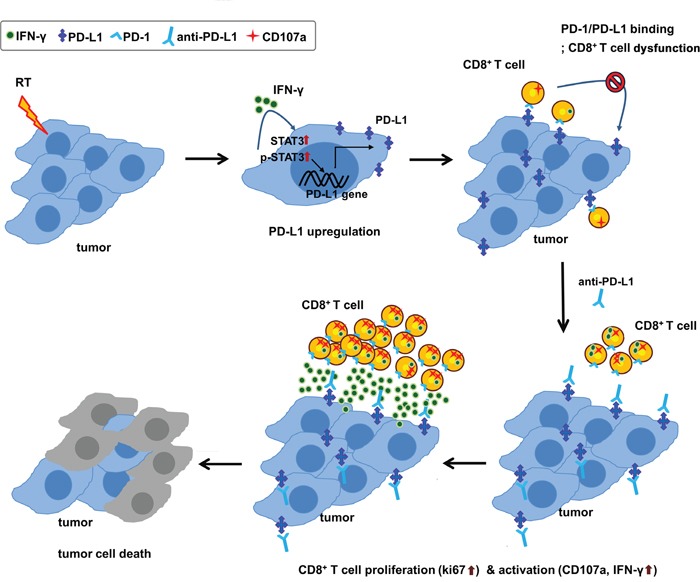
Schematic diagram of proposed mechanism for tumor destruction induced by RT and PD-L1 blockade

In summary, this study demonstrated that radiation led to the upregulation of PD-L1 expression in tumor through increased IFN-γ and enhancing the activity of STAT3, which could facilitate the antitumor effect of anti-PD-L1. The combination of anti-PD-L1 and radiation significantly improved the antitumor effect shown in tumor growth delay as well as in survival. In addition, combination therapy could restore CD8^+^ T cells to expand and regain their effector functions, such as cytokine production and cytolysis (Figure 6). These findings suggest that radiation-induced PD-L1 expression plays a key immunomodulatory action in a combination of anti-PD-L1 and radiation. Our findings provide a strong rationale for future clinical investigation strategies for a combination therapy of anti-PD-L1 and radiation in HCC patients.

## MATERIALS AND METHODS

### Mice

Six- to seven-week-old male C3H/HeN mice were purchased from Orientbio (Seongnam, Gyounggi, Republic of Korea). All mice were maintained in a specific pathogen-free facility of the Yonsei University Medical College guidelines, the Guide for the Care and Use of Laboratory Animals and the Guidelines and Policies for Rodent experiment provided by the Assessment and Accreditation of laboratory Animal Care (AAALAC). All mice were raised with free access to food and water under specific pathogen-free conditions in a room maintained on a 12 h light/dark cycle.

### Cell culture

Murine HCC, HCa-1, was maintained in Dulbecco's minimal essential medium (GIBCO, Carlsbad, CA) supplemented with 10% (v/v) fetal bovine serum (GIBCO), 100 U/ml penicillin and 100 μg/ml streptomycin (GIBCO) at 37°C in a humidified atmosphere of 5% CO_2_ and 95% air.

### Reagents

Cell Fixation/Permeabilization Kits for intracellular cytokine analysis was purchased from BD Bioscience (San Joes, CA). STAT3 inhibitor VI, S31-201 was purchased from Santa Cruz Biotechnology (CA, USA). Recombinant mouse IFN-γ and TNF-α were purchased from Peprotech (Rocky Hill, NJ).

### Real-time RT-PCR

Total RNA was extracted from HCa-1 cells using TRIzol reagent (Life technologies, Carlsbad, CA). One μg of total RNA was reverse transcribed to cDNA using Omniscript RT Kit (Qiagen, Valencia, CA) according to the manufacturer's instructions. Real-time PCR was performed on the Step One Plus-Real Time System (Applied Biosystems, Tokyo, Japan) using the Power SYBR Green Master Mix (Applied Biosystems). The primers were as follows: mouse PD-L1;5’-AAA TCG TGG TCC CCA AGC-3’ and 5’-TCC TCA TGT TTT GGG AAC TAT CT-3’, mouse INF-γ; 5’-CCT AGC TCT GAG ACA ATG AAC GCT-3’ and 5’-TGC CAG TTC CTC CAG ATA TCC AAG-3’, mouse TNF-α;5’-AAA ATT CGA GTG ACA AGC CTG TAG-3’ and 5’-CCC TTG AAG AGA ACC TGG GAG TAG-3’, mouse STAT1; 5’- TGA GAT GTC CCG GAT AGT GG-3’ and 5’-CGC CAG AGA GAA ATT CGT GT-3’, mouse STAT3; 5’-GTC TGC AGA GTT CAA GCA CCT-3’ and 5’-TCC TCA GTC ACG ATC AAG GAG-3’, mouse IRF-1; 5’-GGA CTC AGC AGC TCT ACC CTA CCT-3’ and 5’-GCT GGA GTT ATG TCC CTT TCC ATA TC-3’, and mouse GAPDH; 5’-CGA CTT CAA CAG CAA CTC CCA CTC TTC C-3’ and 5’-TGG GTG GTC CAG GGT TTC TTA CTC CTT-3’. The relative levels of mRNA were determined by normalization of the data to the expression levels of GAPDH mRNA.

### Western blot

Whole cell extracts were prepared using RIPA buffer (Cell Signaling Technology, Beverly, MA) with protease inhibitor cocktail (Roche, Switzerland). Equal amounts of cell lysates were separated on 10% SDS–polyacrylamide gel, and were then electrotransferred to polyvinylidene difluoride membrane (GE Life sciences, Pittsburgh, PA). The membranes were blocked with 5% skim milk in 0.1% TBS-Tween20 for 1 h at 25°C prior to incubation with primary antibodies against PD-L1 (Proteintech, Rocky Hill, NJ), IFN-γ (Bioss, Woburn, MA), TNF-α (Santa Cruz Biotechnology), phospho-STAT1 (S727) (Cell Signaling Technology, Beverly, MA), STAT1 (Cell Signaling Technology), phospho-STAT3 (Y705) (Santa Cruz Biotechnology), STAT3 (Santa Cruz Biotechnology), and GAPDH (Santa Cruz Biotechnology). Primary antibodies were incubated for overnight at 4°C after blocking. For antibodies detection, goat-anti-rabbit IgG-HRP antibody (Santa Cruz Biotechnology) was incubated for 1 h at 25°C. The blots were developed using chemiluminescent substrate (Thermo Fisher Scientific Inc., Waltham, MA) and Signals were detected using ImageQuant LAS-4000 (Fujifilm, Tokyo, Japan). GAPDH was used as a loading control.

### Animal experimental design and X-ray irradiation

HCa-1 cells (1 × 10^6^) were inoculated intramuscularly into the right thighs of mice, and treatment was started when the tumor reached to 8 mm in mean diameter. Four experimental groups were set; control, anti-PD-L1 alone, radiation alone, and combination of anti-PD-L1 and radiation. Mice were immobilized in specially designed mice jig and lead shield was used to avoid unwanted irradiation to other body parts. The right thighs of the mice were irradiated with 10 Gy in 1 fraction using an X-Rad 320 irradiator (Precision X-ray, North Branford, CT). Mice were treated 69 cm from the radiation source (SSD) with a dose rate of 150 cGy/min with 300 kVp X-rays, using 12.5 mA and an X-ray beam filter consisting of 2.0 mm Al. For the PD-L1 blockade experiment, anti-PD-L1 (10 mg/kg, Bio X Cell, West Lebanon, NH) was given as a total of 4 injections in 3-day intervals after radiation. For CD8^+^ T cell depletion experiment, anti-CD8 (12.5 mg/kg, Bio X Cell) was given in a total of 4 injections in 3-day intervals. Antibody treatment started from one day before radiation.

### Tumor growth experiments

Tumor volume was measured every 3 days with an electronic caliper and calculated as volume = π/6 × ab^2^, where ‘a’ is the long axis and ‘b’ is the short axis of two orthogonal diameters. The maximum allow able size of tumors in mice is 20 mm in diameter according to the IACUC (Institutional Animal Care and Use Committee) guidelines of the Yonsei University Health System. After experiments, the experimental mice were sacrificed before reaching the maximum allowable size using carbon dioxide (CO_2_).

### Assessment of survival rate

Mice were monitored daily to ensure their survival rate for 56 days period. When tumor reached to ≥ 25 mm in mean diameter and/or mice showed signs of poor body condition including respiratory distress, hypoactivity, and failure to respond to stimuli, the mice were sacrificed by CO_2_ inhalation and the date was recorded to calculate survival rate.

### TIL preparation

For the isolation of TILs, solid tumors were excised and chopped by clipper then incubated in 1 mg/ml collagenase type IV (Worthington, Lake wood, NJ) solution containing 0.01 mg/ml DNase I (Sigma, CA, USA) at 37°C for 30 min. TILs were isolated by density gradient centrifugation on an 80%/40% Percoll (GE Life sciences) gradient after washing the dissociated tissues by chilled complete RPMI medium.

### Flow cytometry analysis

Flow cytometry was performed by FACS LSR II (BD Bioscience, San Joes, CA). The data was analyzed using the FlowJo software (Tree Star Inc, San Carlo, CA).

For staining of surface proteins, single cells were resuspended in cold PBS with 3% BSA and stained with a fluorescent-labeled antibodies against PD-L1 (ebioscience, San Diego, CA) and CD8 (BioLegend, San Diego, CA) at a final concentration of 1 μg/mL for 15 min at 4°C on ice/light protected. Cells were then washed twice and immediately analyzed.

For intracellular molecule staining, single cells were stained using Cell Fixation/Permeabilization Kits, according to the manufacturer's instructions. Briefly, surface stained cells were fixed and permeabilized for 30 min in a fixation/permeabilization buffer. After washing with 1× Perm/Wash buffer, cells were stained with fluorescent-labeled antibodies against IFN-γ (ebioscience), TNF-α (BioLegend), p-STAT3 (Y705) (ebioscience), CD107a (ebioscience), and Ki67 (ebioscience) for 30 min on ice/light protected. Cells were then washed twice with 1× Perm/Wash buffer and immediately analyzed.

### Histology

For paraffin sections, tumor samples were fixed in 4% formalin and were embedded in paraffin, which was cut into 5-μm-thick sections. For frozen sections, formalin-fixed tumor samples were snap frozen over liquid nitrogen in a Tissue-Tek OCT compound (Sukura, Finetechnical, Tokyo, Japan) and cryosectioned (15 μm). The antibodies used in this study are described in Supplementary Table 1.

For IHC staining, the deparaffinized sections were treated with 3% H_2_O_2_ in methanol for 30 min in order to reduce endogenous peroxidase activity. They were blocked with 5% normal horse serum (Gibco) for 1 h and then incubated with a primary antibody against PD-L1 (Proteintech). The samples were incubated with biotinylated secondary antibody (DAKO Corp., Carpinteria, CA) and peroxidase-labeled streptavidin (DAKO Corp.). Staining was developed using the 3-3 diaminobenzidine substrate chromogen system (DAKO Corp.). The samples were counterstained with hematoxylin (DAKO Corp.) and imaged by light microscopy.

For immunofluorescence staining, frozen sections were incubated with 5% normal horse serum and 0.3% Triton X-100 (Sigma) in PBS, pH 7.5, and subsequently with primary antibodies against CD8 (Abcam, Cambridge, MA), IFN-γ (Bioss), CD107a (Abcam), and Ki67 (Abcam) overnight at 4°C. Finally, sections were incubated with alexa Fluor 488 donkey anti-rabbit IgG (Invitrogen, Carlsbad, CA,) and alexa Fluor 594 goat anti-rat IgG (Invitrogen) at 25°C for 1 h. The sections were then counterstained with DAPI and imaged by confocal microscopy (Carl Zeiss Co. Ltd., Oberkochen, Germany).

### TUNEL assay

Tumor apoptosis was assessed by TUNEL staining. About 5 μm sections of tumor tissues embedded in paraffin were stained with the DeadEnd™ Colorimetric TUNEL System (Promega, Madison, WI), according to the manufacturer's instructions.

### Statistical analysis

Data are expressed as means ± standard error (SE). Statistical significance was determined via one-way ANOVA using Prism 5.0 software (Graphpad Software, La Jolla, CA). A 2-tailed Student's t test was used to compare data between 2 groups. Two-way ANOVA was used to compare tumor size at multiple time points within groups. Differences in mouse survival rate were determined by a log-rank (Mantel-Cox) test of the Kaplan-Meier survival curves. A value of *P*< 0.05 was regarded as statistically significant. All experiments were conducted at least twice.

## SUPPLEMENTARY MATERIALS FIGURES AND TABLE



## Abbreviations

HCC: Hepatocellular carcinoma
ICB: immune checkpoint blockade
CTLA-4: cytotoxic T lymphocyte-associated antigen-4
PD-1: programmed death 1
RT: radiation therapy
PD-L1: programmed death–ligand1
TIL: Tumor-infiltrating lymphocyte
TUNEL: terminal deoxynucleotidyl transferase dUTP nick end labeling
IHC: immunohistochemistry
IRF-1: interferon regulatory facoter-1
