# Supplementary material for: Radiation improves antitumor effect of immune checkpoint inhibitor in murine hepatocellular carcinoma model
Source: Oncotarget. 2017 Apr 17;8(25):41242–55. doi: 10.18632/oncotarget.17168 (PMC5522235; doi:10.18632/oncotarget.17168)
Supplement: Supplementary file 1 [file oncotarget-08-41242-s001.pdf]

Radiation improves antitumor effect of immune checkpoint inhibitor in murine hepatocellular carcinoma model

SUPPLEMENTARY MATERIALS AND METHODS

Orthotopic model

Orthotopic model: Mice of orthotopic models were anesthetized with an intramuscular injection of Zoletil® and Rompun® mixture. An incision was made in the right aspect of anterior abdominal wall, and 5×10^5 HCa-1 cells were injected into the right lobe of livers. To minimize leakage of tumor cells in the abdominal cavity, injection was performed slowly and the injection site was covered with Surgicel® after removal of needle. At about 14 days after implantation of tumor, small animal magnetic resonance imaging (MRI, Bruker BioSpec 94/20 USR, Bruker Biospin, Ettlingen, Germany) was performed to check the development, growth, and location of the tumor. Following that, mice were anesthetized and immobilized in supine position with adhesive tape. The right side of liver was irradiated by 1.5×1.5 cm^2 field, and lead shields were used to prevent irradiating other body parts such as bowel or lung. Mice were sacrificed at day 7 after radiation.

siRNA transfection

Cells were transfected according to the instruction for transfection on lipofectamin (Invitrogen). HCa-1 cells were cultured to 50% confluence in 6-well plate. Cells were transfected with the control, STAT1, or STAT3 siRNA (Santa Cruz Biotechnology) using Lipofectamine 2000, in accordance with the manufacturer's recommendations. Briefly, in each well, siRNA and 2 µl Lipofectamine were separately diluted in 100 µl serum-free medium, mixed, and incubated at room temperature for 20 min. Then, the cells were refreshed with serum-free media after removal of the regular supplemented media. siRNA-Lipofectamine complexes were added to each dish containing cells. Plate was incubated at 37°C for 4 h to allow transfection to occur, and then growth media containing 10% serum was added. After 48 h of additional incubation, cells were treated with 10 Gy radiation.

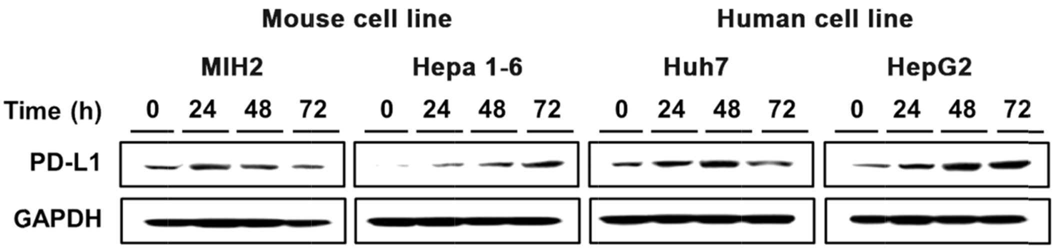

Supplementary Figure 1: Radiation increased expression of PD-L1 in murine and human HCC cells. HCC cells were treated with 10 Gy radiation for the indicated times. Increased expression of PD-L1 was measured by western blotting. GAPDH was the loading control. Data were derived from three independent experiments.

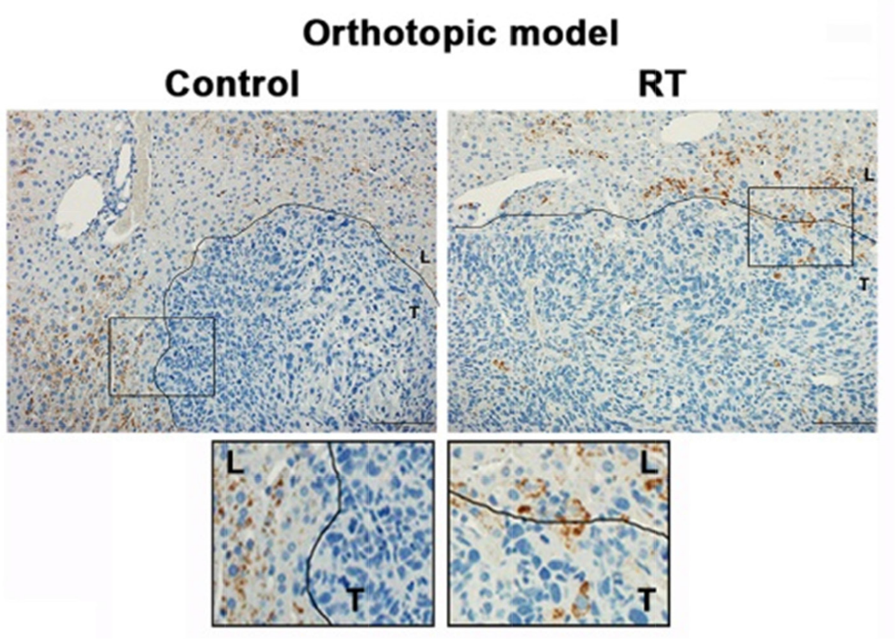

Supplementary Figure 2: The effect of radiation on PD-L1 expression *in vivo* was measured in orthotopic model; mice implanted with HCa-1 cells were treated with 10 Gy radiation, and PD-L1 expressions were assessed in tumors and adjacent liver to the tumor tissue which was obtained after 7 days, by IHC staining (original magnification 200×, scale bar = 100 µm, L = liver, T = tumor).

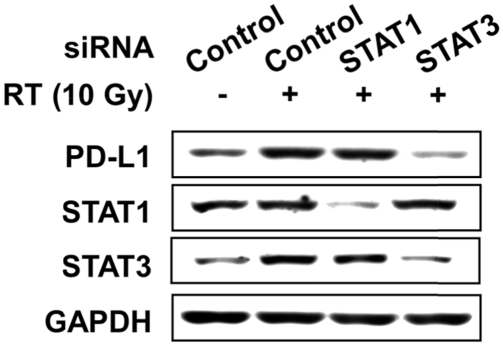

Supplementary Figure 3: HCa-1 cells were transfected by the indicated siRNAs. At 48 hours after transfection, cells were treated by 10 Gy radiation for 48 h, and immunoblotting was performed for PD-L1, STAT1, and STAT3. GAPDH was the loading control. Data were derived from three independent experiments.

Supplementary Table 1: Antibody list

Antigens	Manufacturer	Application
PD-L1	Proteintech, Rocky Hill, NJ	WB, IHC
IFN-γ	Bioss, Woburn, MA	IF
CD107a	Abcam, Cambridge, MA	IF
Ki67	Abcam, Cambridge, MA	IF
CD8	Abcam, Cambridge, MA	IF

Abbreviations:

IHC, immunohistochemistry; IF, immunofluorescence
